# Lung Cancer Cell-Derived Exosome Detection Using Electrochemical Approach towards Early Cancer Screening

**DOI:** 10.3390/ijms242417225

**Published:** 2023-12-07

**Authors:** Koosha Irani, Hossein Siampour, Abdollah Allahverdi, Ahmad Moshaii, Hossein Naderi-Manesh

**Affiliations:** 1 Department of Biophysics, Faculty of Biological Sciences, Tarbiat Modares University, Tehran P.O. Box 14115-154, Iran; kosha.irani@modares.ac.ir (K.I.); naderman@modares.ac.ir (H.N.-M.); 2 Biosensor Research Center (BRC), Isfahan University of Medical Sciences, Isfahan P.O. Box 81746-73461, Iran; h_siam@modares.ac.ir; 3 Department of Physics, Tarbiat Modares University, Tehran P.O. Box 14115-175, Iran; moshaii@modares.ac.ir

**Keywords:** exosomes, early lung cancer diagnosis, electrochemical biosensor, A549 cell line

## Abstract

Lung cancer is one of the deadliest cancers worldwide due to the inability of existing methods for early diagnosis. Tumor-derived exosomes are nano-scale vesicles released from tumor cells to the extracellular environment, and their investigation can be very useful in both biomarkers for early cancer screening and treatment assessment. This research detected the exosomes via an ultrasensitive electrochemical biosensor containing gold nano-islands (Au-NIs) structures. This way, a high surface-area-to-volume ratio of nanostructures was embellished on the FTO electrodes to increase the chance of immobilizing the CD-151 antibody. In this way, a layer of gold was first deposited on the electrode by physical vapor deposition (PVD), followed by thermal annealing to construct primary gold seeds on the surface of the electrode. Then, gold seeds were grown by electrochemical deposition through gold salt. The cell-derived exosomes were successfully immobilized on the FTO electrode through the CD-151 antibody, and cyclic voltammetry (CV) and electrochemical impedance spectroscopy (EIS) methods were used in this research. In the CV method, the change in the current passing through the working electrode is measured so that the connection of exosomes causes the current to decrease. In the EIS method, surface resistance changes were investigated so that the binding of exosomes increased the surface resistance. Various concentrations of exosomes in both cell culture and blood serum samples were measured to test the sensitivity of the biosensor, which makes our biosensor capable of detecting 20 exosomes per milliliter.

## 1. Introduction

Lung cancer is the second most common cancer in humans and the leading cause of cancer death globally, with about one million deaths annually [1]. Based on statistics provided by the World Health Organization, the number of patients and deaths is almost comparable, indicating the late diagnosis of this cancer. As a result, the treatments are useless in those stages that cause the patients to die. On the other hand, its initial symptoms, such as coughing, shortness of breath, and weight loss, are present in many diseases that require a complicated cancer diagnosis. Chest radiography, PET scans, and bronchoscopy are conventional methodologies for diagnosing lung cancer. All of them are based on imaging and can detect a tumor or cancerous tissue when its diameter reaches at least 1 cm, and it is highly likely to metastasize and invade other tissues. In addition, these methods are directly or indirectly harmful to the patient [2,3]. Thus, finding ways based on biomarkers for early diagnosis is necessary. Much attention has been paid to biomarkers such as microRNAs, exosomes, and tetraspanin proteins that can help us obtain information about intracellular events. Exosomes are nano-sized vesicles secreted by most cells and contain crucial information about their source cells. Exosomes have a lipid bilayer membrane on the surface, containing marker proteins originating from the secreted cell [4]. They are commonly detected in the presence of mobile waste products, along with nucleic acids, proteins, and transmembrane proteins, including CD63, CD81, CD9, TSG101, and CD151 [5]. These reveal physiological and pathological changes in the cell or tissue of their origin. Furthermore, they can be found in various body fluids, including plasma, malignant ascites, urine, amniotic fluid, and saliva. These features highlight the exosome as a potential marker for diagnosis. Studies have shown that cancer cells release larger amounts of extracellular vesicles to transform non-metastatic cells into metastatic ones [6]. Recent investigations have indicated that extracellular vesicles released by cancer cells alter the environment around the tumor and are implicated in the transformation of stromal cells into angiogenic cells, pre-metastatic cells, or tumor-inhibiting cells [7]. Exosomes released from cancer cells have many tumor antigens on their surface, which can be utilized in the non-invasive early detection of cancer and monitoring of the treatment process [8]. Therefore, over the last decade, several detection structures have been developed for the quantitative evaluation of exosomes, including strategies based on the nanoparticle tracking analysis method (NTA), western bolt, fluorescence, surface plasmon resonance (SPR), surface-enhanced Raman spectroscopy (SERS), and electrochemical sensing [9]. Among these, electrochemical detection is particularly active thanks to its advantages of simple operation, quick response, high specificity, and high accuracy [10,11,12]. Electrochemical approaches have become increasingly important in biomedicine in recent years. Electrochemistry is the study of the relationship between electrical and chemical processes, and it has a wide range of applications in biomedicine. One area where electrochemical approaches are particularly useful is the development of biosensors. Biosensors are analytical devices that use biological components to detect and measure specific chemical compounds or biological molecules. Electrochemical biosensors use electrodes to measure changes in electrical signals that occur when a biological molecule binds to a specific receptor on the electrode surface. This approach has been used to develop biosensors for various applications, including glucose monitoring for diabetes, detection of infectious diseases, and monitoring of environmental pollutants [13,14]. Accurate and quantitative measurement of exosomes at very low concentrations is vital in biochemical analysis. Conventional approaches are time-consuming and require large exosomes isolated from blood or culture medium. Therefore, developing a reasonable and accurate biosensor for cancer screening is necessary. Electrochemical techniques with features such as very high sensitivity, ease of use, and requires a small amount of sample can be used to make biosensors with high accuracy [15]. Among the various techniques that exist in the electrochemical system, cyclic voltammetry (CV) and electrochemical impedance spectroscopy (EIS) methods were used in this project. The basis of the CV technique is establishing the electric potential in the system at a specific interval and checking the electric current created by these changes in penance. The range of changes is selected according to the electrolyte used in the system, whose value indicates the oxidation and reduction points of the elements in the electrolyte. In the EIS method, a small potential is applied to the sample as a variable with time, and the impedance of the system is determined. Due to their high sensitivity (in the range of microvolts and ohms) in measuring surface changes, these two techniques are a very suitable option for making sensors to identify biomarkers with low concentrations in the sample. In addition, integrating nanotechnology with electrochemistry can increase the sensitivity of this method for measuring biomarkers [16,17]. Various materials, such as conductive polymers, metals, semiconductors, etc., can make electrochemical biosensors [18]. To increase the signal and sensitivity of electrochemical sensing systems, it is important to find a material that can be used as a surface with properties such as small size, large electroactive surface area, good biocompatibility, and suitable conductivity. Gold nanostructures are an excellent alternative for making electrochemical biosensors because of their remarkable electrical properties, easy interconnection of different functional groups, and excellent biocompatibility [19]. In this regard, a noteworthy point is the change in gold’s electrical and catalytic properties according to the size and shape of its particles [20]. As a result, using an objective and reliable approach for the deposition of the gold layer is very important. Employing the physical vapor deposition (PVD) technique next to electrodeposition is an excellent choice to acquire the expected properties due to the ability to control the size and shape of particles. In addition, the surface must be such that there is adequate space for the probe to connect. In other words, increasing the surface area by volume by providing more space can increase the value of the biosensor.

**Figure 1:**
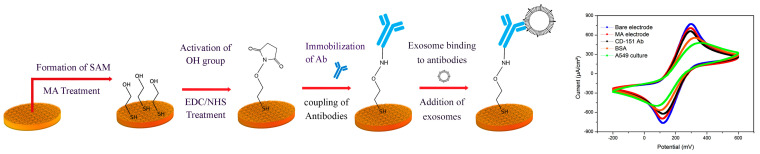
Creation of functional groups to stabilize antibodies on the electrode and then connect surface antigens of exosomes to antibodies.

In this project, an attempt has been made to develop a reliable and reproducible, sensitive biosensor based on determining the number of exosomes using electrochemical methods to detect and determine the number of exosomes. For this purpose, it was done first with the appropriate layer of gold, followed by thermal annealing to construct primary gold seeds on the surface of the electrode. Then, these primary gold seeds grew to the appropriate size and activated the surface by creating functional groups with high-performance materials while observing the required times for each step. In the following steps, the design of system control tests was evaluated, and its capability was confirmed. To validate the biosensor, exosomes derived from cancer cells and human serum as a real sample are used. The schematic diagram of the different stages of this project is shown in Figure 1, which can be seen from the stages of surface activation and attachment of antibodies and then exosomes to them, which causes a change in the passing current and electrode resistance.

## 2. Results

### 2.1. Examination of Exosomes by Staining with DPH

**Figure 2:**
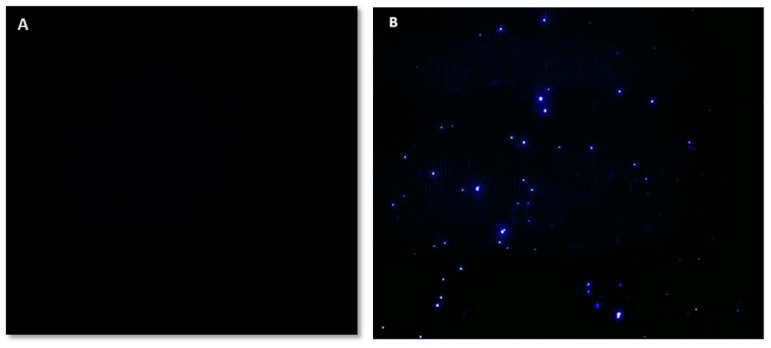
A DPH stain with the property of binding to phospholipid and emitting fluorescent light was used to witness the presence of an exosome in the sample. (**A**) Fluorescent image of an exosome-free (negative control) sample (10× magnification). (**B**) Fluorescent image of a DPH-stained exosome sample (10× magnification).

To check the presence of exosomes in the environment, the fluorescent dye DPH, which can bind to the phospholipid membrane of exosomes, was used. DPH dye, with its hydrophobic and fluorescent properties, is one of the most suitable options for staining vesicles with two membrane layers in such a way that in solutions containing membrane vesicles with an aqueous solvent, this dye penetrates the membrane and is placed between two membrane layers. In the next step, with a tool such as a fluorescent microscope, we detect the light emitted by this dye, which indicates the presence of vesicles in that part. In this way, some dye was added to the sample and photographed with a fluorescence microscope. As seen in Figure 2, the bright spots indicate the binding sites of DPH and the lipid membrane.

### 2.2. Exosome Size Measurement by DLS

**Figure 3:**
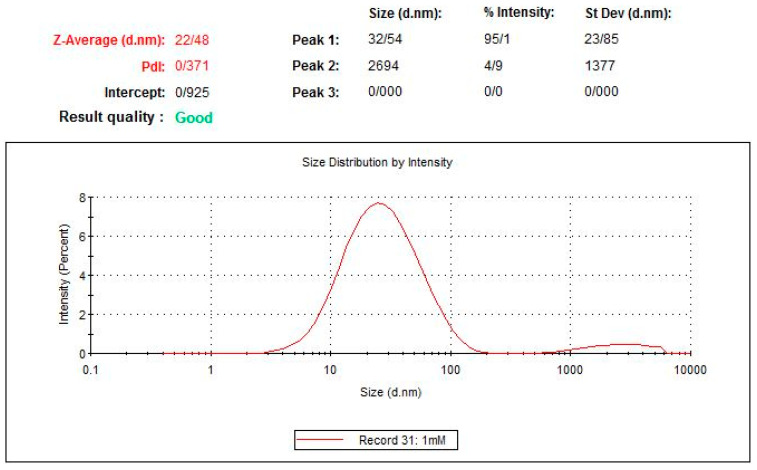
The diagram of measuring the size of exosomes by DLS shows a bell-shaped size distribution with a peak of about 32/54 nm, and the polydispersity index value (PDI) is 0/371.

Dynamic light scattering is one of the most accurate, fast, repeatable, and relatively low-cost methods for measuring the hydrodynamic radius and other dynamic characteristics of nanoparticles suspended in a liquid. As can be seen in Figure 3, the range of changes in the size of the particles in the sample is about 35 nm for the population of extracted exosomes, which shows the efficiency of the ultracentrifugation method in removing other particles and extracting the pure exosome.

### 2.3. Exosome Investigation with AFM

**Figure 4:**
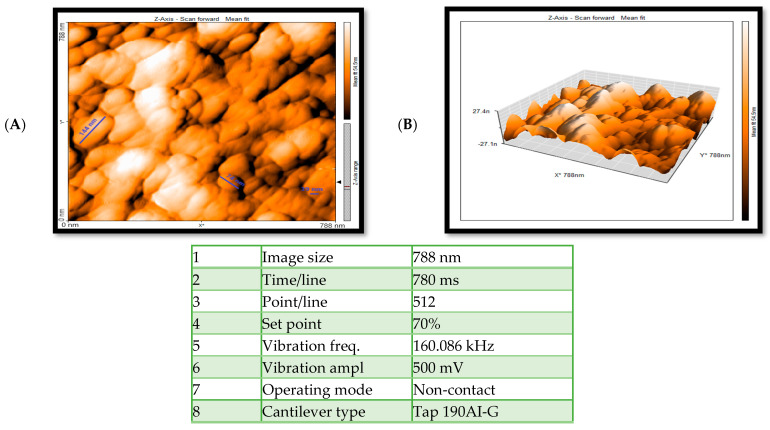
The images obtained from the atomic force microscope show exosomes of different sizes. The two-dimensional image of exosomes shows that the largest exosome seen in the image is 144 nm in diameter, and the smallest exosome is 39 nm in diameter. In the three-dimensional image of exosomes, the lows and highs are visible. It is clear. (**A**) 2D AFM image of exosomes immobilized on slides. (**B**) 3D AFM image of exosomes immobilized on slides.

The images obtained from the atomic force microscope show exosomes of different sizes. Figure 4A shows the two-dimensional image of exosomes, whose diameter is about 40 nm. Figure 4B is a three-dimensional image of exosomes with clearly defined heights.

### 2.4. Characterization of Exosomes with the SEM Electron Microscope

**Figure 5:**
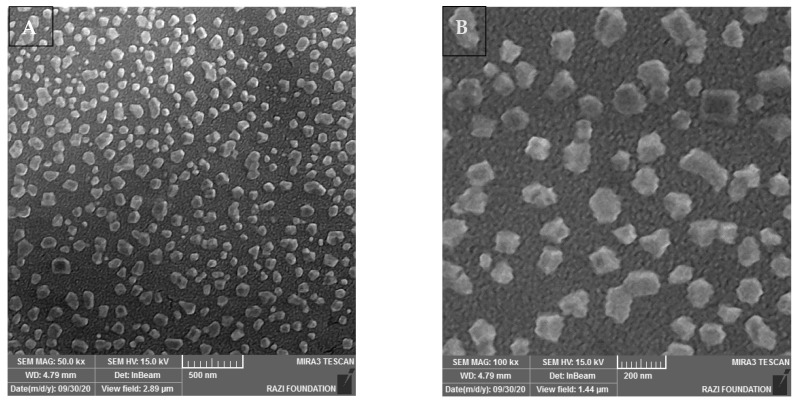
Electron microscope images of exosome samples. The average size of the particles is about 50. The reason for the larger size and shape of the particles is due to their coating. (**A**) Image with 50 kx magnification. (**B**) Image with 100 kx magnification.

One of the most accurate methods to check the size and shape of the exosome is the electron microscope, which provides an accurate image of the sample with high magnification power and an appropriate detection limit. Figure 5 shows exosomes are well purified and have the right size. The reason for their non-spherical nature is the changes that occur during the process of dehydrating and stabilizing them.

### 2.5. Quantification of the Number of Exosomes by Flow Cytometry

**Figure 6:**
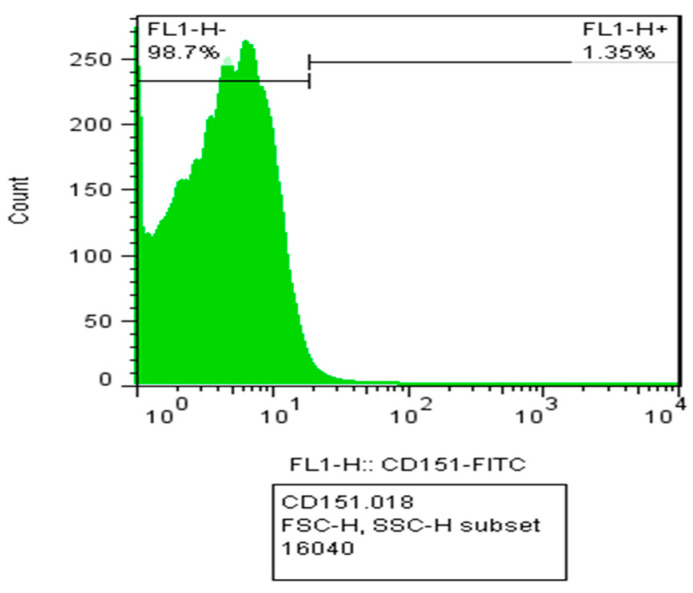
Flow cytometry diagram to calculate the number of exosomes by CD151-labeled antibody.

The purpose of flow cytometry here is to determine the number of exosomes per unit volume and the relationship between the number of exosomes and electrochemical parameters. As it is clear in Figure 6, there are about 16,040 particles in the tested sample, and about 1.35 percent of them are attached to the labeled antibody, which indicates the presence of 216 exosomes in the environment.

### 2.6. Lung Cancer Exosome Biosensor Assembly

#### 2.6.1. Seed-Mediated Gold-Nanostructure

By reviewing research and articles, we can understand the efficiency and importance of using gold to modify FTO surfaces. The surface of FTO electrodes has been modified to reach the highest surface area to volume ratios, bond an increased number of capture probes, and enhance the electrochemical performance through the fabrication of gold nano-islands (AuNI) on the surface. By using nanostructures with a high surface area-to-volume aspect ratio, we could immobilize more antibodies on the surface, enhancing the sensitivity of the biosensor. 

Our findings showed that by using the Au-nanostructure seed-mediated strategy, lung cancer exosomes could be successfully detected. Liu Z. et al. [21] used DNA/ferrocene-modified single-walled carbon nanotube complexes under optimal experimental conditions. They could detect the exosomes of A549 cells with high accuracy (detection limit of 9.38 × 10^4^ exosomes/mL). Compared with their methods, our Au-nanostructure design is more sensitive and capable of detecting exosomes at 20/mL, which is very important for early cancer diagnosis applications.

#### 2.6.2. Electrode Preparation

To use the electrodes as biosensors, it is necessary to go through several steps of layering and activation so that the first FTO electrode is applied. Next, the nanostructure (through the seed modification method) was grown on the FTO electrodes. A 5 nm-thick gold layer was deposited by the physical vapor deposition (PVD) approach, followed by electroplating in a HAuCl_4_ (0.5 mm) solution. Then we pour MA solution on the electrodes, and this material is connected to the gold coated on the surface in the previous processes and causes the formation of an OH group. Then, by adding EDC/NHS, which are dissolved in the MES buffer. These substances are connected to OH, and creating an NH group makes the surface ideal for antibody binding. By going through these steps, the gold nanostructure gets a prominent structure that increases the effective area/volume ratio, thus increasing the immobilizing efficiency of CD-151 antibodies. The result demonstrated the high conductivity and chemical stability of nanostructured electrodes. The CD-151 antibody was immobilized on the surface of the gold nanostructure and bound to the surface by the chemical NH group. In the next step, to block the active surface and nonspecific binding, a drop of bovine serum albumin (BSA) was added to the surface of the nanostructure. By performing CV and EIS tests and comparing the changes to the antibody immobilization step, it can be understood that the surface was blocked. In the last step, complementary hybridization of the CD-151 surface antigens of exosomes with antibodies was done through hydrogen bonding. At each phase, electrochemical tests, including EIS and CV curves, were performed.

The assembled biosensor improved electrochemical signals based on the high surface area compared to the aspect ratio of seed-mediated Au-nanostructure. Furthermore, it may improve the sensitivity and selectivity of electrochemical biosensors. The assembled biosensor provides synergetic effects of high sensitivity, conductivity, specificity, and biocompatibility.

### 2.7. Electrochemical Assays of Gold Nanostructure

**Figure 7:**
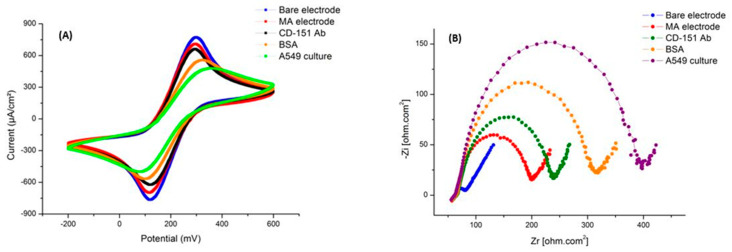
Exosome concentration tests using the CD151 antibody in an electrochemical system. (**A**) The CV diagram shows the changes in the current with each layering step. In the last step, the binding of exosomes to the antibody caused a decrease in the current. (**B**) EIS diagram showing changes in surface resistance with each layering step. In the last step, the binding of exosomes to antibodies increased the resistance.

In the first step, we performed the experiment on the culture medium collected from A549 cells, which was purified by several steps of centrifugation. Electrochemical changes in the experiment were monitored through CV and EIS testing at each phase. The relevant graphs are presented in Figure 7. The CV and EIS are effective electrochemical characterization parameters that confirm the proper manufacture of the gold nanostructure, immobilization of the capture probe, and hybridization of the exosome. These tests were carried out using a redox probe of [Fe(CN)_6_]^4−^/[Fe(CN)_6_]^3−^. Electrochemical characterization was performed in the potential range of −0.3 to 0.6 V. This figure shows the CV diagrams of the electrode in bare mode, the MUA binding step, the EDC/NHS connection to the surface, the immobilization of antibodies, the addition of BSA solution, and the stage of adding antigen. By comparing the results, you can be sure of the proper binding of the antibody to the antigens. In this figure, the same results of the CV test can be seen in the EIS charts. The changes in EIS data at different stages indicate an increase in the potential peak.

In addition, the binding of antibodies and antigens has increased the resistance of the electrode surface and decreased the rate of electron transfer. The diameter of the semicircle part of the EIS Nyquist plots shows the electron-transfer resistance (R_et_), and the linear element in the low frequency corresponds to the diffusion-limited process. The AuNS bare FTO shows a R_et_ value of around 5 Ω (ohm). By connecting the ME, the surface resistance reaches 50 Ω. When the CD-151 antibody is immobilized on the FTO AuNSs electrode, R_et_ increases to 90 Ω. Then, BSA solution was added, and the diameter changed to 140 Ω. The exosome and antibody were then hybridized, causing the R_et_ to rise to 200 Ω.

**Figure 8:**
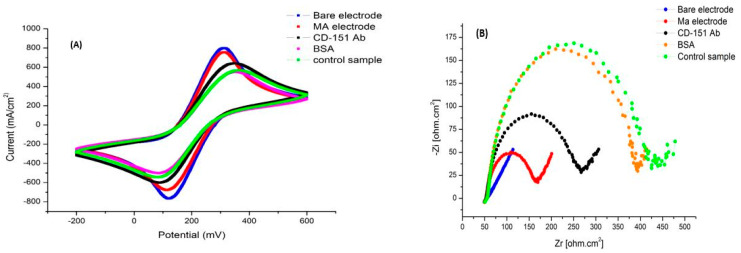
Electrochemical diagrams related to the test medium without an exosome. (**A**) The CV diagram shows the changes in the current with each layering step. In the last step, due to the absence of exosomes, there was no change in the current. (**B**) EIS diagram showing changes in surface resistance with each layering step. In the last step, due to the absence of exosomes, there was no change in resistance.

In the next step, to make sure that the binding of exosomes and antibodies caused changes, a culture medium without exosomes was used. As it is clear in Figure 8, the changes in CV and EIS diagrams can be seen in different stages as in the previous test, except in the last stage, which indicates the specificity of the antibody and the lack of binding to other factors in the environment.

**Figure 9:**
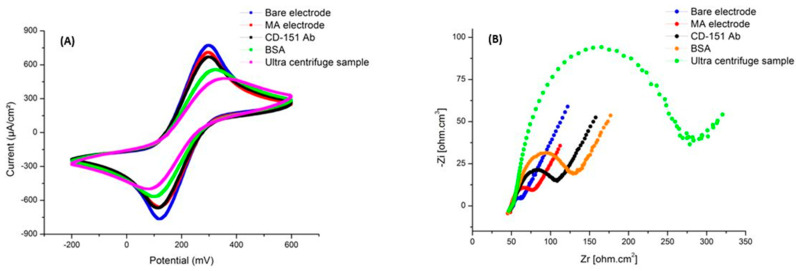
Electrochemistry test results with an ultracentrifuged sample. (**A**) The CV diagram shows the changes in the surface current with each layering step. In the last step, due to the high purity and high amounts of exosomes, a significant change in the current was created. (**B**) EIS diagram showing changes in surface resistance with each layering step. In the last step, due to high purity and high amounts of exosomes, a significant change in resistance has been created.

In the continuation of the experiment, we used an ultracentrifuge at 100,000 rpm to obtain more accurate results for exosome purification and then repeated the steps as in the previous tests. As can be seen in Figure 9, the change in the stage of adding antigen is several times that of the previous experiment, which indicates the binding of more exosomes to the antibodies on the electrode surface, a greater increase in resistance, and, as a result, a greater reduction in electron transfer.

**Figure 10:**
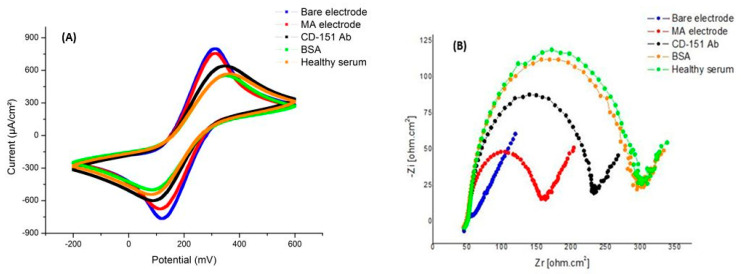
Electrochemistry test results with healthy serum. (**A**) The CV diagram shows the changes in the current with each layering step. In the last step, due to the absence of exosomes, there was no change in the current. (**B**) EIS diagram showing changes in surface resistance with each layering step. In the last step, due to the absence of exosomes, there was no change in resistance.

In the final stage, we investigated the ability of the biosensor to immobilize exosomes inside the serum. In this phase of the experiment, the serum sample of a healthy person was used as an antigen. Therefore, all the steps were carried out like the previous tests, and only in the final step was the surface of the electrode covered with diluted serum, and then the tests for CV and EIS were taken. As can be seen in the graphs in Figure 10, there is not much change in the resistance and current of the electrode in the phase of adding serum, which can be concluded because the antibodies fixed on the surface have not established a connection with anything in the serum.

**Figure 11:**
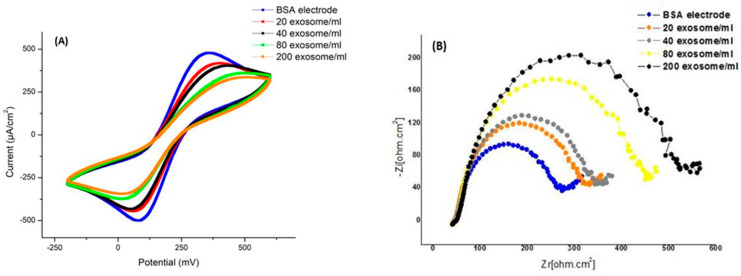
Electrochemistry test results with various concentrations of exosomes. (**A**) CV diagram showing changes in surface current at different concentrations of exosomes. As the concentration increases, the current decreases. (**B**) EIS diagram showing changes in surface resistance at different concentrations of exosomes. The resistance increases with increasing concentration.

In the next step, the sensitivity of the biosensor was checked with different concentrations of exosomes. In this way, we added concentrations including 1/100 (20 exosomes per mL), 1/50 (40 exosomes per mL), 1/25 (80 exosomes per mL), 1/10 (200 exosomes per mL), and 1 to the electrode. After the appropriate time passed, we took CV and EIS tests from them. As can be seen in Figure 11, the biosensor’s ability to detect exosomes is about 20 particles per mL.

**Figure 12:**
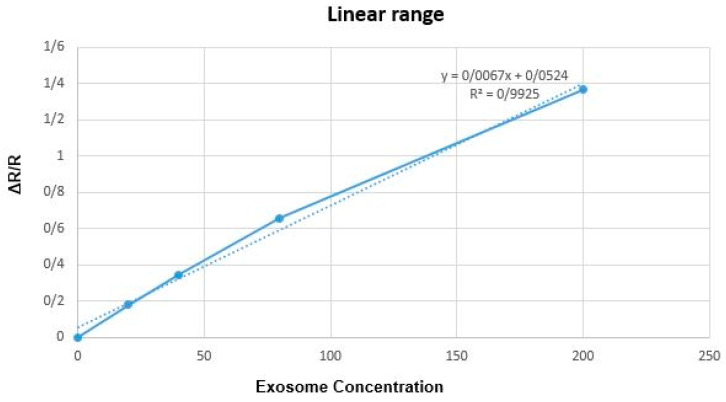
The graph of the relationship between changes in surface resistance and exosome concentration. Resistances at each stage are normalized to baseline (BSA). The resistance in the step of adding BSA is considered the base level (R = 230). Then, the resistance (R) difference in each step is calculated and divided by the base resistance level to be normalized (ΔR/R). Solid line: experimental data, dashed line calculated fitted model.

In the last step, we examined the relationship between changes in surface resistance and changes in exosome concentration so that the surface potential corresponding to different concentrations was normalized concerning the base level in the BSA stage. Then, the diagram in Figure 12 was drawn. As can be seen in the figure, the resistance has changed linearly with increasing concentration.

## 3. Discussion

Lung cancer is one of the most common types of cancer worldwide and one of the deadliest. Several factors contribute to the high mortality rate of lung cancer. One of the main reasons for the high mortality of lung cancer is that it is often not detected until it has reached an advanced stage [22]. This is because early-stage lung cancer usually does not cause any symptoms, and by the time symptoms do appear. The cancer may have already spread to other parts of the body. Therefore, scientists paid attention to biomarkers, either miRNA or exosomes, released by cancer cells into the circular system for early detection [23]. Exosomes are particularly interesting in cancer research because they are released by cancer cells and contain specific biomolecules characteristic of cancer. For example, exosomes released by cancer cells contain DNA mutations, RNA expression patterns, and protein markers associated with cancer development and progression [24]. One of the advantages of using exosomes as cancer biomarkers is that they can be easily isolated from biological fluids such as blood, urine, and saliva. This makes them a non-invasive and readily accessible source of diagnostic information. In addition, because exosomes are released by cancer cells, they can provide information on the molecular characteristics of the tumor itself, which can be useful for tailoring treatment plans [25]. Exosomes are useful biomarkers for various types of cancer, including lung cancer, breast cancer, prostate cancer, and ovarian cancer. However, there are still some challenges associated with using exosomes as cancer biomarkers. One of the main challenges is that exosomes are heterogeneous, meaning they can vary widely in their composition and molecular characteristics. This can make it difficult to identify specific exosome biomarkers characteristic of a particular type of cancer. Our work focused on the exosomes secreted by lung cancer cells (A549). We used the CD-151 antigen in these exosomes to identify them from the rest of the environmental factors. In different stages of this research, several centrifugation stages at different speeds and then an ultracentrifuge stage at high speed were used to purify these exosomes. In addition, the use of the electrochemical technique, which has a very high sensitivity to changes, along with the selection of precise and high-efficiency deposition and layering methods, made the results very accurate.

Kilic et al. has reported an unlabeled electrochemical biosensor designed to measure the release of MCF-7 cell exocystic in the breast cancer cell line. The biosensor is constructed based on the signal modification that occurs when anti-CD81 recognizes CD-81 on the lipid membranes of small extracellular vesicles (EVs) from breast cancer. This biosensor showed a detection limit (LOD) of 77 particles/mL [26]. The steps of surface activation and binding of antibodies and antigens in this research are similar to those in our project, with the difference that we designed the steps of layering and growth of gold to create a more suitable surface for binding. As a result, our obtained limit of detection (LOD) has been imporoved to 20 exosomes/mL.

Sahu et al. used certain surface markers, including tumor-related protein epidermal growth factor receptor (EGFR) and exosome tetrapolymer family proteins of CD9 and CD63, to detect exosomes. Their biosensor provides an LOD of 4.9 × 10^6^ particles/mL [27].

Wang et al. developed an aptasensor assisted by a nanotetrahedron (NTH) to capture and detect exosomes from hepatoma cells directly. Tetrahedral DNA was used to modify the electrode’s initial design to specifically recognize exosomes. The tetrahedral DNA contains the aptamer DNA sequence. The LOD was 2.09 × 10^4^ particles/mL [28].

Li et al. have developed electrochemical impedance spectroscopy to quantify exosome-specific external (tetraspanine) and internal (syntenine) markers. In their study, the detection limit for exosomes was 1.9 × 10^5^ particles/mL [29]. S. Yadav et al. achieved a diagnostic limit of 4.7 × 10^5^ exosomes/µL for breast cancer by using surface antigen CD9 and human epidermal growth factor receptor 2 (HER-2) in the electrochemical system [30]. Doldán et al., used the sandwich method in the form of a gold electrode modified with α-CD9 antibody and HRP-conjugated IgG antibody to detect exosomes. In the first step, the electrode is incubated with the sample so that the exosomes bind to the antibody. Then, the HRP-conjugated IgG antibody is added to the environment to identify α-CD9 and increase the signal. This method showed an LOD of 200 particles/mL [31].

Surfactants and stabilizers are commonly used in electrochemical biosensors to enhance the performance and stability of the sensing interface. However, they can introduce interference, non-specific binding, or electrode fouling, compromising sensitivity and selectivity. By eliminating these additives, surfactant- and stabilizer-free electrochemical biosensors offer improved accuracy and reduced non-specific interactions [32]. One of the advantages of our biosensor is that it is completely surfactant- and stabilizer-free. One of the key advantages of surfactant- and stabilizer-free electrochemical biosensors is their enhanced sensitivity. Surfactants and stabilizers can alter the properties of the electrode surface and the diffusion behavior of analytes, thereby affecting the electrochemical response. By directly interfacing with the analyte without additives, these biosensors can provide more accurate and reliable signal transduction, leading to improved sensitivity [33].

## 4. Materials and Methods

### 4.1. Exosome Isolation from A549 Cells

In this research, A549 cells were used since they are involved in most clinical cases with non-small lung cells (NSCCL) and produce and secrete many exosomes. They are selected as candidates for exosome secretion in an in vitro environment. Cells were purchased from Pasteur Institute Cell Bank (Tehran, Iran) and cultured in DMEM containing 5% exosome-free fetal bovine serum (FBS), 1% streptomycin, and penicillin at 37 °C. The culture medium was collected from the cells after about three days when the cell confluency reached 70%. To separate the exosomes from the culture medium, the medium was centrifuged at 2000× *g* for 10 min at room temperature. The supernatant was centrifuged at 10,000× *g* for 30 min to eliminate large microvesicles like ectosomes and oncosomes. The supernatant resulting from the centrifugation was then ultra-centrifuged at 100,000× *g* for 2 h. After all the centrifugation stages, the collected pellet contained only exosomes and other small particles, like lipoprotein. The collected pellet was resuspended in phosphate-buffered saline for further analysis.

### 4.2. Characterization of Cell-Derived Exosomes

For a general evaluation of the presence, concentration, and size of exosomes, several quantitative and qualitative tests were performed. First, the obtained exosomes were stained with DPH dye to ensure that the particles were of membrane type. Then, we examined the size determination test using dynamic light scattering (DLS), flow cytometry, and examination with AFM and SEM microscopes.

#### 4.2.1. Staining with DPH

DPH dye was used to check the presence of exosomes, which can bind to the phospholipid membrane and have fluorescence properties. In this process, a 1:20 ratio of exosome-rich medium to dye was used to add 50 microliters of DPH dye to a volume of 1 mL of the medium. Then, it was examined and evaluated by an inverted Olympus IX81 fluorescent microscope equipped with a DP-72 camera.

#### 4.2.2. Particle Size Characterization by DLS

The DLS instrument (Malvern Panalytical, Malvern, UK, Nano-ZS) was used to determine the size and distribution of particles in the sample. In this way, about 1 mL of the purified exosome sample was poured into the cuvette of the device and placed inside the device, and the data from the sample was analyzed.

#### 4.2.3. Particle Topography Assessment by AFM

An AFM microscope was used to examine the morphology (surface characteristics) of the exosome. To prepare the sample for photography with this microscope, the desired slide was first washed with acetone and placed at 60 degrees. Then, the slide was completely dried, and 50–70 microliters of pure exosome liquid were poured on the slide and left for 1–2 h at room temperature to dry. Finally, the prepared sample was evaluated by AFM (Nanosurf-CoreAFM, Liestal, Switzerland).

#### 4.2.4. Particle Morphology Assessment by SEM

To analyze the surface topography, the morphology of the sample, the fracture surfaces, etc., images of the exosome sample were prepared with an SEM electron microscope. This research used a field emission electron microscope (FESEM, MIRA3 TESCAN Co., Ltd., Taipei, Taiwan) with a resolution of 1.5 nm at 15 KV voltage and 4.5 nm at 1 KV voltage. It needed a series of preparation steps before placing the sample under the microscope. This way, the sample was dehydrated, fixed to the surface, and coated with gold or platinum.

#### 4.2.5. Particle Quantification by FACS

To calculate the number of exosomes in the sample, the flow cytometry technique was used. The basis of this device is irradiating a laser at a specific wavelength and then classifying and counting the light reflected from the fluorescent sample. In this way, CD151 antibody bound to FITC fluorescent dye was added to the purified exosome sample and measured.

### 4.3. Electrochemical-Based Biosensor Design

#### 4.3.1. Solvents

The following materials were used to carry out this project: FTO glass (Nanogostar Sepahan Company, Isfahan, Iran), autoclaved ultrapure water (MilliQ water, Merck Millipore, Burlington, MA, USA), KCl, Fe^2+^, Fe^3+^, HAuCl_4_, phosphate-buffered saline (PBS), N-ethyl-N′-(3-(dimethylamino)propyl) carbodiimide (EDC), N-hydroxysuccinimide (NHS), bovine serum albumin (BSA), mercaptoacetic acid (MAA), and potassium ferro/ferricyanide electrolyte.

#### 4.3.2. Buffers

The solvent used for aqueous solutions was purified and autoclaved water (MilliQ Water). In the growth stage of gold nanostructures, HAuCl_4_ gold salt with a concentration of 0.5 mM was used. For CV and EIS tests, a standard electrolyte (potassium ferricyanide) was used. The antibody concentration in this project was 5 μg/mL, and PBS buffer was used to dilute the main antibody stock under sterile conditions.

Absolute ethanol was used to prepare the mercaptoacetic acid (MAA) solution. This substance was dissolved in ethanol at a ratio of 1 to 10. MES buffer was used to prepare the EDC/NHS solution, which was prepared by mixing MES (2-Morpholino-ethansulfonsaure) monohydrate and NaCl in distilled water.

#### 4.3.3. Electrochemical Instruments

The system consisted of a cell containing three electrodes: a working electrode (FTO electrode modified by gold nanostructure), a counter electrode (gold foil 20 × 8 mm^2^), and a reference electrode (Ag-AgCl). The steps were as follows: first, gold nanostructures were fabricated and grown on the FTO electrode with the physical vapor deposition and electrochemical deposition methods, and then, in different stages, the cyclic voltammetry (CV) and electrochemical impedance spectroscopy (EIS) tests were taken. All electrochemical measurements were performed with an Origalysis Potentiostat/Galvanostat model OGF01A.

### 4.4. Electrochemical Procedures

#### 4.4.1. Design and Fabrication of Gold Nanostructure

In preparation for use, FTO bare electrodes (20 × 8 mm^2^) were rinsed with the ultrasonication of deuterium-depleted water (DDW) containing soap detergent, absolute acetone, and ethanol successively for 15 min. After several washing steps, the electrodes were placed in a PVD chamber with their FTO side facing upwards to deposit a thin layer of gold. Next, the electrodes were placed in a furnace (at 500 °C for 10 h) so that the gold nanoparticles were firmly fabricated on the surface of the electrodes. Then a 0.5 mM HAuCl_4_ solution (15 min at 0.1 V) was used for electrochemical growth (chronoamperometry) of gold particles. For CV tests, the potential ranges were set between −300 and 600 mV, the scanning speed was 50 mV/s, and the electrolyte included a solution of KCl (0.1 M) and [Fe(CN)_6_]^4−^/[Fe(CN)_6_]^3−^ (2.5 mM), as the redox pair. In addition, for EIS tests, the device was set at the initial frequency of 100 kHz and the final frequency of 100 mHz.

#### 4.4.2. Immobilization of the Antibody on the Electrode

To bind antibodies and perform electrochemical testing, activating the electrode surface by functional group is vital, which ME and EDC/NHS solutions accomplished. First, a 2-ME solution was poured on the electrodes, placed at room temperature for 2 h, and washed. Then EDC/NHS was poured on them and refrigerated at −4 °C for one hour. The CD151 antibody was stored at −20 °C and before for use it was first transferred to −4 °C and then brought to the appropriate concentration by phosphate buffer saline (PBS). After washing the electrodes, the CD151 antibody was applied and refrigerated overnight at −4 °C. In this step, to block the places where the antibody was not attached, BSA solution dissolved in PBS buffer was used by washing the electrodes containing antibodies, pouring BSA on them, and placing them at room temperature for 30 min. Finally, to study the connection of exosomes with antibodies and evaluate the accuracy of the biosensor, CV and EIS examinations were measured at various concentrations of exosomes fixed on the electrodes. To achieve the best efficiency, it is very important to pay attention to the fact that the time interval between activating the surface of the electrodes and their use is minimized.

#### 4.4.3. Exosome Assay in the Real Serum Sample

The biosensor performance analysis was carried out using the standard addition method. To this end, different concentrations of the exosomes were added to real human serum samples (4 times diluted in PBS). The serum was donated by our colleague with ethical approval considerations, according to the instructions of the ethics committee at Tarbiat Modares University. Five different concentrations of exosomes in serum samples, including 1/100 (20 exosomes per mL), 1/50 (40 exosomes per mL), 1/25 (80 exosomes per mL), 1/10 (200 exosomes per mL), and 1/1 (2000 exosomes per mL), were tested. All measurements, such as EIS and CV, were performed in at least three replicates.

## 5. Conclusions

Lung cancer often goes undetected in its early stages, as symptoms may not manifest until later stages of the disease. Common symptoms include persistent coughing, chest pain, shortness of breath, wheezing, coughing up blood, unexplained weight loss, and fatigue. Early detection through screening tests like biomarkers screening can also significantly improve outcomes by identifying lung cancer at an earlier, more treatable stage. Exosomes are small extracellular vesicles that play important roles in intercellular communication and are potential biomarkers for various diseases, including cancer. The detection and analysis of exosomes hold great promise for early disease diagnosis, prognosis, and monitoring of treatment responses. Electrochemical methods have emerged as powerful tools for the sensitive and selective detection of exosomes due to their unique advantages. Electrochemical detection offers several benefits for exosome analysis. Firstly, electrochemical sensors and biosensors provide excellent sensitivity, allowing the detection of low concentrations of exosomes in complex biological samples. This sensitivity is attributed to the amplification effects of electrochemical signals and the use of nanomaterials with high surface area-to-volume ratios. Secondly, these methods offer rapid detection with real-time or near-real-time results. Electrochemical assays often involve simple sample preparation and straightforward measurement procedures, making them suitable for point-of-care applications. Thirdly, electrochemical techniques are compatible with miniaturization and integration into portable devices, enabling on-site analysis and facilitating remote monitoring of disease progression.

Particularly in our tailor-made biosensor, an LOD of 20 exosomes/mL in spiked blood serum was obtained, which is good and has application for further cancer screening. While electrochemical methods for exosome detection show great potential, there are still challenges to overcome. These include further improvements in sensitivity, selectivity, and reproducibility, as well as the standardization of sample preparation protocols and the validation of results across different platforms. Additionally, careful consideration is required for proper validation and standardization of biomarkers associated with exosomes to ensure accurate and reliable diagnosis and monitoring of diseases.

## Data Availability

The raw data will be available upon reasonable request to the corresponding author.
